# Transplantation of tissue-engineered human corneal endothelium in cat models

**Published:** 2013-02-18

**Authors:** Tingjun Fan, Xiya Ma, Jun Zhao, Qian Wen, Xiuzhong Hu, Haoze Yu, Weiyun Shi

**Affiliations:** 1 Key Laboratory for Corneal Tissue Engineering, Ocean University of China, Qingdao, Shandong Province, China; 2 Key Laboratory of Ophthalmology, Shandong Eye Institute, Shandong Academy of Medical Sciences, Qingdao, Shandong Province, China

## Abstract

**Purpose:**

To evaluate the performance of reconstructed tissue-engineered human corneal endothelium (TE-HCE) by corneal transplantation in cat models.

**Methods:**

TE-HCE reconstruction was performed by culturing 1,1'-dioctadecyl-3,3,3',3'-tetramethylindocarbocyanine perchlorate (DiI)-labeled monoclonal HCE cells on denuded amniotic membranes (dAMs) in 20% fetal bovine serum-containing Dulbecco’s Modified Eagle’s Medium/Ham’s Nutrient Mixture F12 (1:1) medium and 5% CO_2_ at 37 °C on a 24-well culture plate. The reconstructed TE-HCE was transplanted into cat corneas via lamellar keratoplasty with all of the endothelium and part of Descemet’s membrane stripped. Postsurgical corneas were monitored daily with their histological properties examined during a period of 104 days after transplantation.

**Results:**

The reconstructed TE-HCE at a density of 3,413.33±111.23 cells/mm^2^ in average established intense cell-cell and cell-dAM junctions. After lamellar keratoplasty surgery, no obvious edema was found in TE-HCE-transplanted cat corneas, which were transparent throughout the monitoring period. In contrast, intense corneal edema developed in dAM-transplanted cat corneas, which were turbid. The corneal thickness gradually decreased to 751.33±11.37 μm on day 104 after TE-HCE transplantation, while that of dAM eye was over 1,000 μm in thickness during the monitoring period. A monolayer of endothelium consisting of TE-HCE-originated cells at a density of 2,573.33±0.59 cells/mm^2^ attached tightly to the surface of remnant Descemet’s membrane over 104 days; this was similar to the normal eye control in cell density.

**Conclusions:**

The reconstructed TE-HCE was able to function as a corneal endothelium equivalent and restore corneal function in cat models.

## Introduction

Human corneal endothelial (HCE) cells of the innermost layer of the cornea play vital roles in maintaining corneal transparency by barrier and pump functions. While they are arrested in the G_1_ phase of the cell cycle and do not show proliferation in vivo [1], even primate corneal endothelial cells have been proved to have proliferative capacity in vitro [2]. When HCE cells are damaged due to various causes (e.g., viral infection, intraocular surgery, or Fuchs dystrophy), corneal endothelial dysfunction occurs. Currently, such damage can be repaired only with corneal transplantation [3-5], although a specific Rho/Rho kinase inhibitor has potential for the treatment of corneal endothelial dysfunction [6]. Unfortunately, a very limited number of healthy donor corneas are available for corneal transplantation worldwide [7].

At present, tissue-engineered (TE)-HCE has evolved into an ideal equivalent to traditional donor cornea [8]. Transplantation of HCE cells cultured on carriers such as animal corneal stromata [9], gelatin membranes [10], amniotic membranes (AMs) [11], collagen sheets [12], and collagen-chondroitin sulfate foams [13] in animal models has met with success. Unfortunately, the cells used in these trials originated from oncogene-transfected [14,15], primary cultured [8,16], or limited subcultured (4–5 generations) [9,11] HCE cells. Recently, we have established an untransfected HCE cell line, without any tumorigenicity, from donated corneas [17], and made it possible for these cells to be used as seeder cells in large-scale TE-HCE reconstruction. Since the untransfected HCE cell line exhibited chromosomal aneuploidy, monoclonal HCE cell strains with normal diploid karyotypes and gene expression profile are believed to be the ideal seeder cells for TE-HCE reconstruction. Several monoclonal cell strains with a normal karyotype and gene expression profile upon characterization have been screened out from the untransfected HCE cell line by limited dilution techniques in our laboratory [18], prompting us to hypothesize that these cells can be used for TE-HCE reconstruction and clinical transplantation trial as well.

The rabbit has been proved to be an exceptional mammal whose corneal endothelial cells proliferate even during adult phase [19,20]. Reconstructed TE-HCE functions normally in transplanted eyes of New Zealand white rabbits [21,22]. However, whether it functions alone or in cooperation with inherent corneal endothelial cells is not clear. To verify the function of reconstructed TE-HCE, we reconstructed a novel TE-HCE by using one monoclonal strain of HCE cells and human denuded AM (dAM) and evaluated its structural property and in vivo performance in cat models in this study.

## Methods

### Animals

All cats, male and 2.0–2.5 kg in bodyweight, purchased from Shandong Eye Institute of Shandong Medical Academy (Qingdao, China), were used in accordance with the guidelines in the Association for Research in Vision and Ophthalmology Statement for the Use of Animals in Ophthalmic and Vision Research. Their care and usage for lamellar keratoplasty (LKP) surgery was approved by the Institutional Animal Care and Use Committee of the Shandong Eye Institute.

### In vitro reconstruction of tissue-engineered human corneal endothelium

One strain of monoclonal HCE (mcHCE) cells (designated as C3B), screened from an untransfected HCE cell line established previously in our laboratory [17,18], was propagated in 20% (v/v) fetal bovine serum–Dulbecco’s Modified Eagle’s Medium/Ham’s Nutrient Mixture F12 (1:1) medium (pH 7.2; Dulbecco’s Modified Eagle’s Medium /F12, Invitrogen, Carlsbad, CA). Logarithmic C3B mcHCE cells were harvested with 0.25% trypsin (Sigma-Aldrich, St. Louis, MO) digestion methods, as described previously [21]. Briefly, after the old culture medium was removed, the cells were digested with 0.5 ml of 0.25% trypsin for 1 min. Then the trypsin was removed and the mcHCE cells were suspended in 5 ml Dulbecco’s Modified Eagle’s Medium /F12 medium by pipetting. After cell number counting, 3.22×10^6^ mcHCE cells were precipitated at 1,500 r/min for 10 min, labeled with 2 ml of 10 μg/ml 1,1'-dioctadecy1–3,3,3′,3′-tetramethylindocarbocyanine perchlorate (DiI, Sigma-Aldrich) solution at 37 °C for 5 min and 4 °C for 15 min successively, washed twice with PBS and resuspended in 1 ml 20% (v/v) fetal bovine serum–Dulbecco’s Modified Eagle’s Medium /F12 medium (pH 7.2; Invitrogen). Furthermore, dAMs were prepared from fresh human AMs according to the method described previously [17]. The cells were inoculated into the wells of a 24-well culture plate with dAMs attached at the bottom and cultured in a 5% CO_2_ incubator at 37 °C, with medium refreshed entirely every 3 days as described previously [17]. The morphology and growth status of cells were monitored daily.

### Characterization of the tissue-engineered human corneal endothelium

The cell number was counted using eyepiece graticules with the cell density averaged on five graticules after TE-HCEs were reconstructed for 96 h. The morphology of cell sheets was examined with an inverted microscope (Eclipse TS100; Nikon, Tokyo, Japan) and a JSM2840 scanning electron microscope (SEM; JEOL, Tokyo, Japan). The cell junction was described after being stained with 1% (w/v) alizarin red. The continuous monolayer of HCE cells was confirmed histochemically by freeze section and hematoxylin and eosin (H&E) staining. The attachment of cell sheets to dAMs was examined with a H700 transmission electron microscope (TEM; Hitachi, Tokyo, Japan).

### Tissue-engineered human corneal endothelium transplantation

Six adult cats were divided randomly into two groups (three animals each) for LKP surgery. The right eyes of cats in TE-HCE group were transplanted with TE-HCEs, while those in dAM group were transplanted with dAMs. The left eye of each cat was used as the control (normal control eye). LKP surgery was performed following the methods described previously with minor modifications [7]. The cat were anesthetized with intramuscular 1% (w/v) ketamine hydrochloride (Wuhan Jiuan Pharmaceutical Inc., Wuhan, China) and 2.5% (w/v) chlorpromazine hydrochloride (Hangzhou Minsheng Pharmaceuticals, Hangzhou, China). After disinfection and sterile draping of the operation site, 400 μl of 1% (w/v) pilocarpine and 2.5 kU/ml heparin sodium solution (1:1) were injected into the anterior chamber. The central cornea was trephined using a 7.0-mm diameter Hessburg-Barron trephine (Suzhou Mingren Products, Suzhou, China). Cat corneal endothelium on the corneal button was totally stripped along with part of Descemet’s membrane (DM) using forceps.

A sheet of TE-HCE (cellular side up) was placed onto the denuded posterior surface of the cat corneal button. After reposition, the cat cornea was sutured with a combination of 12 interrupted sutures using 10–0 nylon (Mani, Tochigi, Japan). After 200 μl of aqueous humor was injected into the anterior chamber, the protruding edge of TE-HCE was cut off carefully. Immediately after operation, 0.5 ml ophthalmic ointment (Alcon-Couvreur S.A./N.V., Puurs, Belgium) of 0.3% (w/v) tobramycin and 0.1% (w/v) dexamethasone (1:1) was instilled. In addition, 0.5 ml tobramycin-dexamethasone ophthalmic ointment and 0.1 ml 1% (w/v) prednisolone acetate ophthalmic suspension (Allergan Pharmaceuticals, Mayo, Ireland) were instilled into the conjunctival sac once a day after surgery. Each suture was removed when its nylon thread loosened.

### In vivo examination of cat corneas

Each cornea of the operated eyes and controls was examined in vivo at an interval of 6 days after transplantation. The corneal transparency of each operated eye and control eye was examined with a KJ5D slit-lamp biomicroscope (Kangjie Medical Instrument, Suzhou, China). Corneal thickness was measured using a SW-1000P ultrasound pachymeter (Souer Electronic Technology, Tianjin, China) with the average calculated from 10 readings. Eye pressure was measured with an applanation tonometer (TONO-PEN AVIA^®^, Reichert, NY).

### In vitro examination of transplanted corneas

Cats were sacrificed with an overdose intravenous injection of pentobarbital sodium (Sigma-Aldrich) after deep anesthesia with ether. Corneas were excised from each cat eye on day 104 after transplantation. After being rinsed twice with PBS, each cornea was cut equally across the center into four pieces. The first piece was first examined under a Nikon Eclipse Ti fluorescent microscope for DiI fluorescence as a whole mount sample, and then was stained with 1% alizarin red and examined under a Nikon E200 microscope. The number of corneal endothelial cells was counted using eyepiece graticules with the cell density calculated and averaged based on five random graticules. The second piece was sampled to make paraffin sections and stained with H&E for histological examination using a Nikon E200 microscope. The third piece was sampled for morphological examination using a JSM-2840 SEM (JEOL). Finally, the last piece was sampled for ultrastructural examination using an H700 TEM (Hitachi).

### Statistical analysis

Data were expressed as mean±standard deviation (SD; n=3) and analyzed for statistical significance with one way analysis of variance.

## Results

### Characterization of the in vitro reconstructed tissue-engineered human corneal endothelium

**Figure 1:**
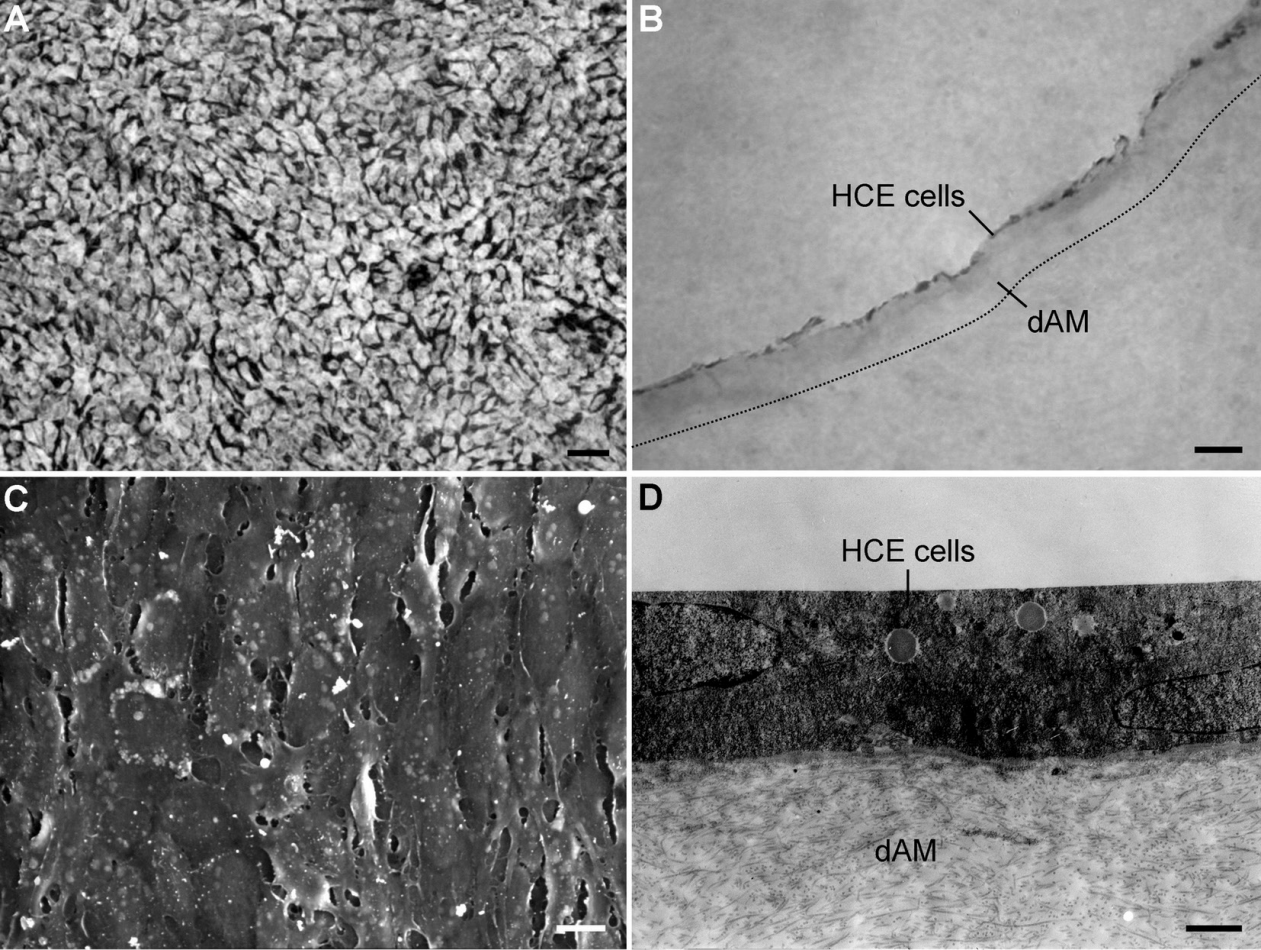
The tissue-engineered human corneal endothelium reconstructed from monoclonal human corneal endothelium (HCE) cells and denuded amniotic membrane (dAM) at day 4. The morphology and structure of tissue-engineered (TE)-HCE cells are shown in 1% alizarin red staining **A**. Frozen sections were stained with hematoxylin and eosin (H&E), **B** and visualized using scanning electron microscopy (SEM) **C** and transmission electron microscopy (TEM) **D**. Scale bars, **A** 20 μm; **B** 100 μm; **C** 10 μm; **D** 2 μm.

On light microscopic examination of the reconstructed TE-HCE, cultured mcHCE cells from C3B strain attached bluntly to dAM, which adhered on the well bottom of a 24-well culture plate, 4 days after inoculation. The mcHCE cells were almost all in polygonal morphology and a confluent monolayer of the cells formed with continuous intercellular junctions established (Figure 1A). The average density of the monolayer sheet was calculated to be 3,413.33±111.23 cells/mm^2^. Histochemically, H&E staining showed that the mcHCE cells formed a confluent monolayer on dAM (Figure 1B). Under SEM, a great majority of the cells were found to be in a polygonal shape (Figure 1C). Under TEM, it was observed that the monolayer of the cells attached tightly to dAM, which were similar to that of HCE cells in vivo (Figure 1D).

### In vivo characterization of cat corneas after tissue-engineered human corneal endothelium transplantation

**Figure 2:**
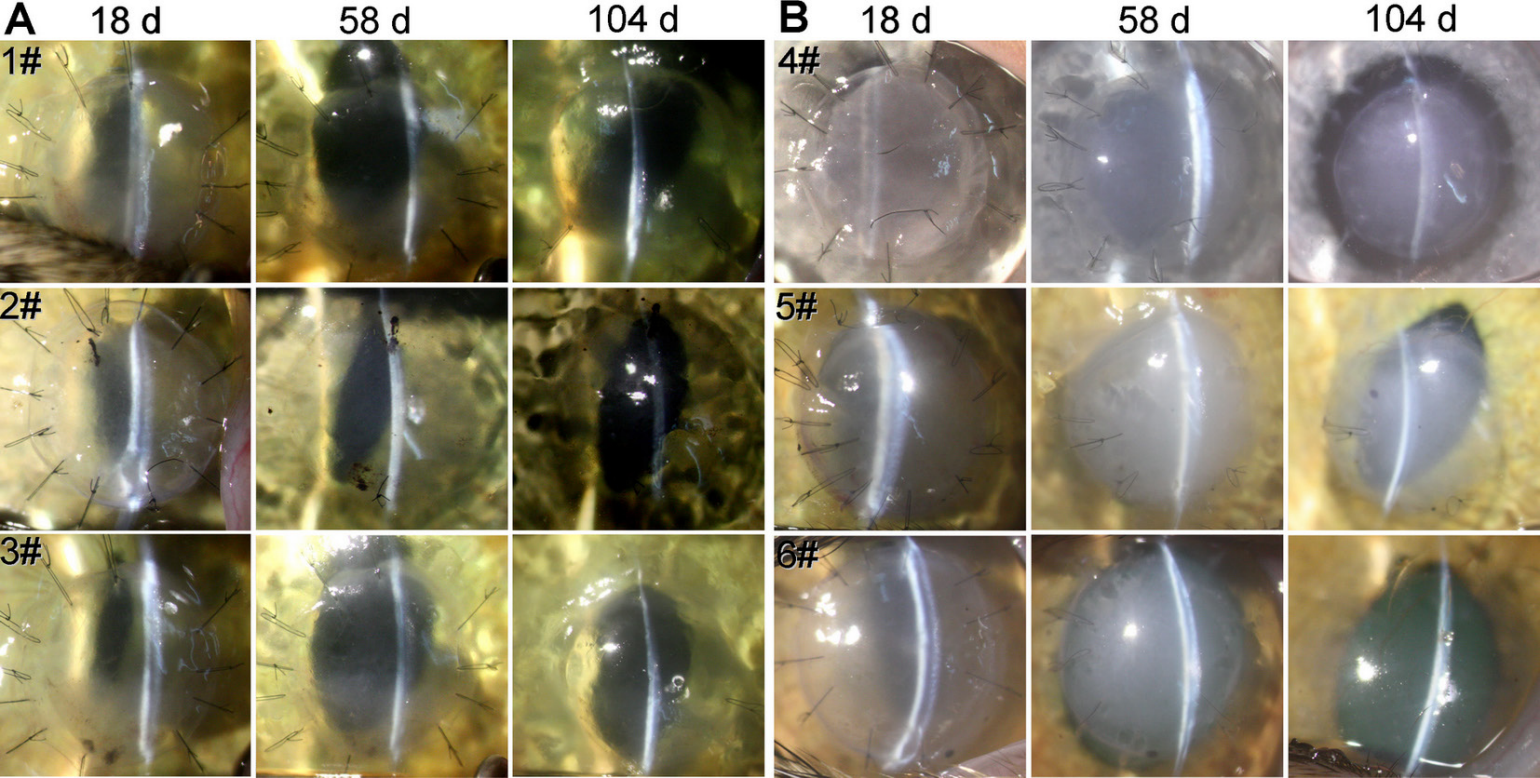
Slit-lamp biomicroscopic photographs of corneas from transplanted cats. The transparency and edema status of cat corneas in the tissue-engineered human corneal endothelium (TE-HCE) group **A** and denuded amniotic membrane (dAM) group **B** at 18, 58, and 104 days after surgery, respectively.

After TE-HCE transplantation, intense corneal edema was found within 30 days after surgery in the TE-HCE group; furthermore, corneal edema decreased much faster in the TE-HCE group than in the dAM group. Representative corneal photographs in each cat on day 18, 58, and 104 after surgery are shown in Figure 2. As compared with the opaque cornea in the dAM group, the cornea transplanted with TE-HCE was clear.

**Figure 3:**
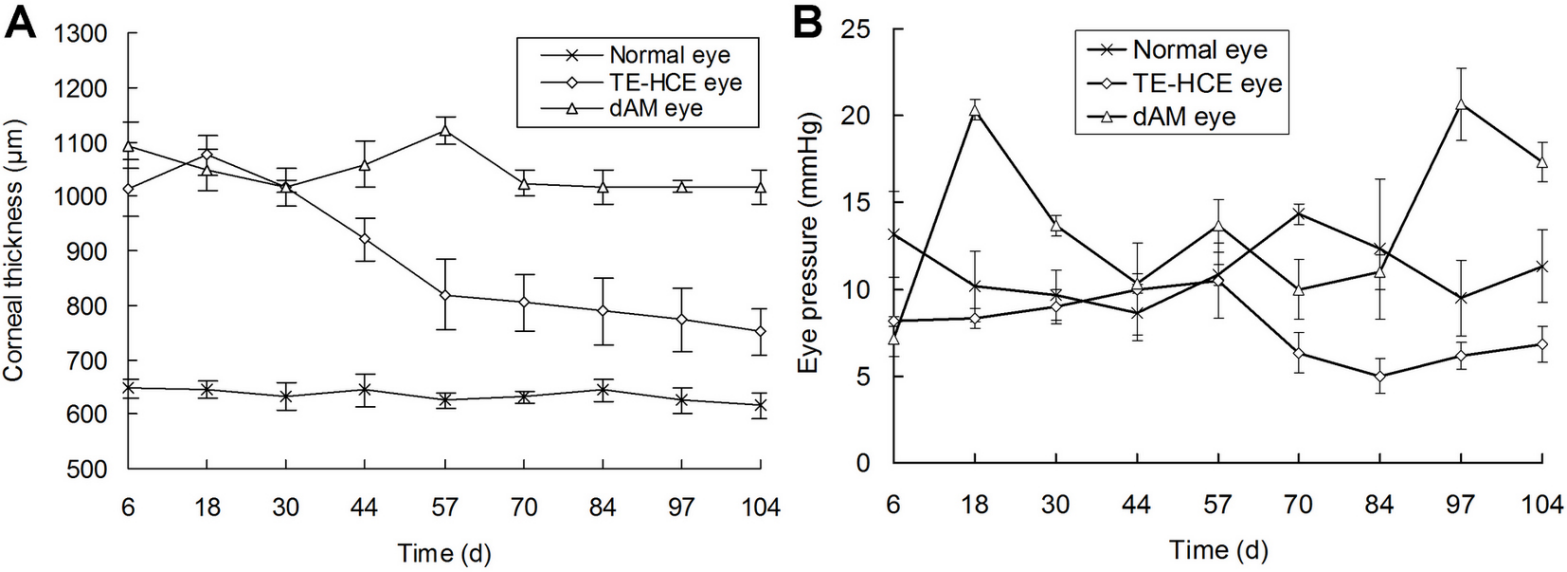
Time-course of average central corneal thickness **A** and the eye pressure **B** of transplanted cats.

The mean central corneal thickness gradually decreased to 751.33±11.37 μm, as was observed on day 104 after transplantation, which was significantly less than that of dAM eyes measured on day 44 (p<0.01; one way analysis of variance test; Figure 3A). In contrast, the corneal transplant was edematous and the average central corneal thickness was over 1,000 μm throughout the observation period of 104 days in the dAM group. The mean corneal thickness remained at 1,015.67±30.92 μm on day 104. Besides, there was no obvious relationship in the eye pressure of TE-HCE, dAM, and normal control eyes, which fluctuated with time (Figure 3B).

### In vitro characterization of cat corneas after tissue-engineered human corneal endothelium transplantation

**Figure 4:**
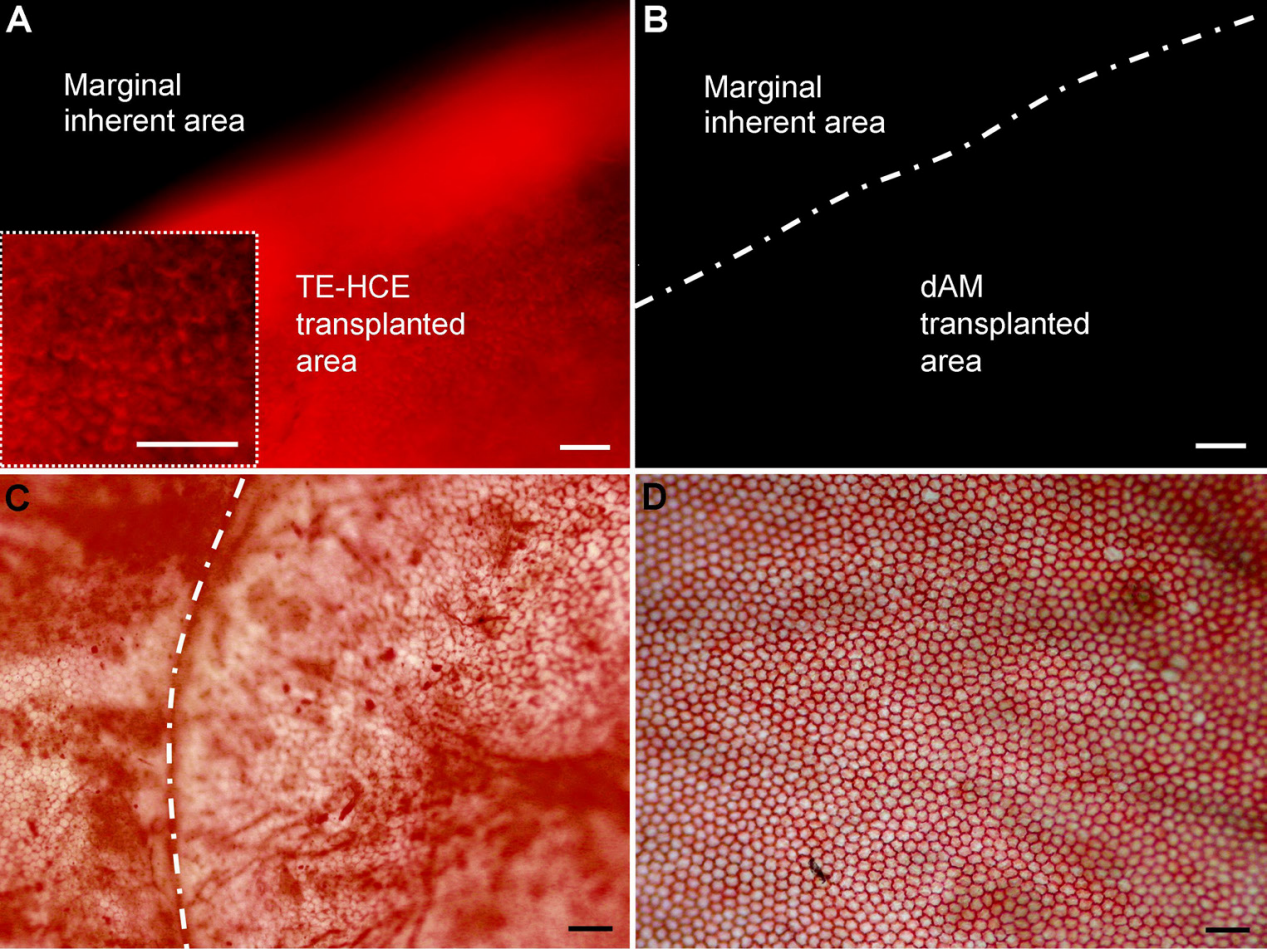
Corneal endothelia from transplanted cats. Fluorescent photographs of 1,1'-dioctadecyl-3,3,3',3'-tetramethylindocarbocyanine perchlorate (DiI)-labeled monoclonal human corneal endothelium (HCE) cells in tissue-engineered (TE)-HCE eye **A** and denuded amniotic membrane (dAM) eye **B** alizarin red staining photographs of the monoclonal HCE cells in TE-HCE eye **C** and normal control eye **D** are shown. In the TE-HCE eye **A**, a cell-visible high magnification view of the cornea is shown in the box with a dashed line. Scale bars **A**, **B** 100 μm; **C**, **D** 50 μm.

On day 104 after surgery, the cells in the transplanted areas of the corneal endothelia of TE-HCE group emitted DiI fluorescence (Figure 4A), while those of the corneal endothelia of dAM and normal control eyes did not (Figure 4B). Some dark areas are even present within the grafted region in Figure 4A, and low levels of fluorescence significantly above background are still present. In addition, the entire margin of the TE-HCE-transplanted areas adjacent to the inherent host cornea emitted high levels of DiI fluorescence. Since DiI fluorescence could only be observed in the mcHCE cells labeled by DiI fluorescent dye, taken together, it can be speculated that the corneal endothelial cells within the TE-HCE transplanted area are all from TE-HCEs, not from migrated host endothelial cells. Abundant intercellular junctions were established and a monolayer of continuous mcHCE cells, either hexagonal or polygonal in shape, was reconstructed in the TE-HCE group (Figure 4C), similar to those of the normal control eyes (Figure 4D). The contour line of the small areas in Figure 4C are feint graphs formed by alizarin red dye accumulation and the endothelial cells inside were stained weakly. The average cell density of the corneal endothelia of TE-HCE eyes was 2,573.33±0.59 cells/mm^2^, also similar to that of the normal control eyes (2,693.33±0.70 cells/mm^2^).

**Figure 5:**
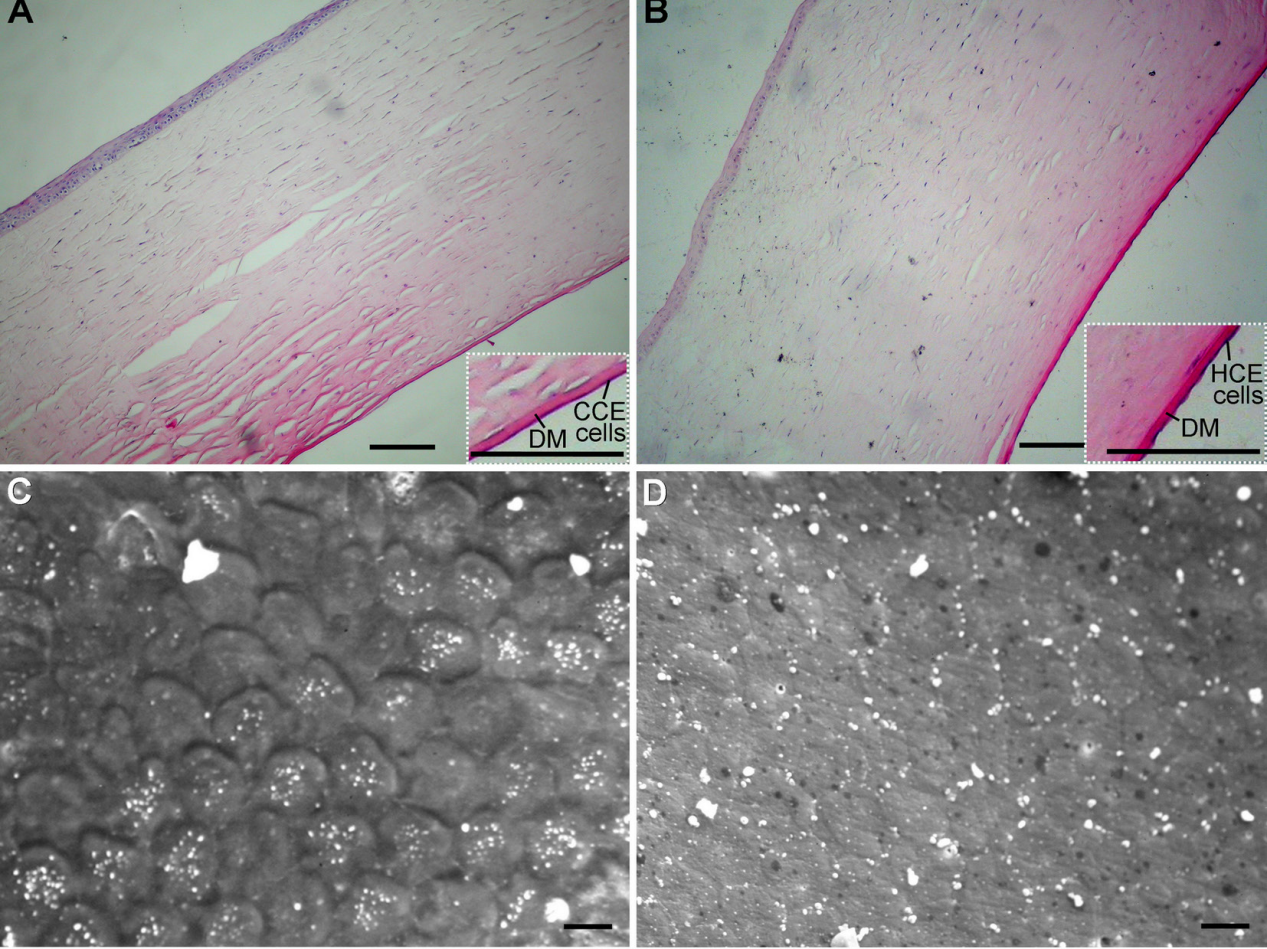
Histological structures of corneas from transplanted cats. Representative corneal photomicrographs of hematoxylin and eosin (H&E) staining in tissue-engineered human corneal endothelium (TE-HCE) eye **A** and normal control eye **B** SEM in TE-HCE eye **C** and normal control eye **D** are shown. Scale bars **A**, **B** 100 μm; **C**, **D** 10 μm.

One hundred and four days post transplantation, a monolayer of continuous cells was formed on the endothelial surfaces of the corneas of the TE-HCE eyes (Figure 5B), and the histological structure and thickness of the corneas were almost the same as those of the normal control eyes (Figure 5A). Under SEM, the mcHCE cells of the TE-HCE eyes were found to be either hexagonal or polygonal in morphology (Figure 5D), almost identical to those of the normal control eyes (Figure 5C).

**Figure 6:**
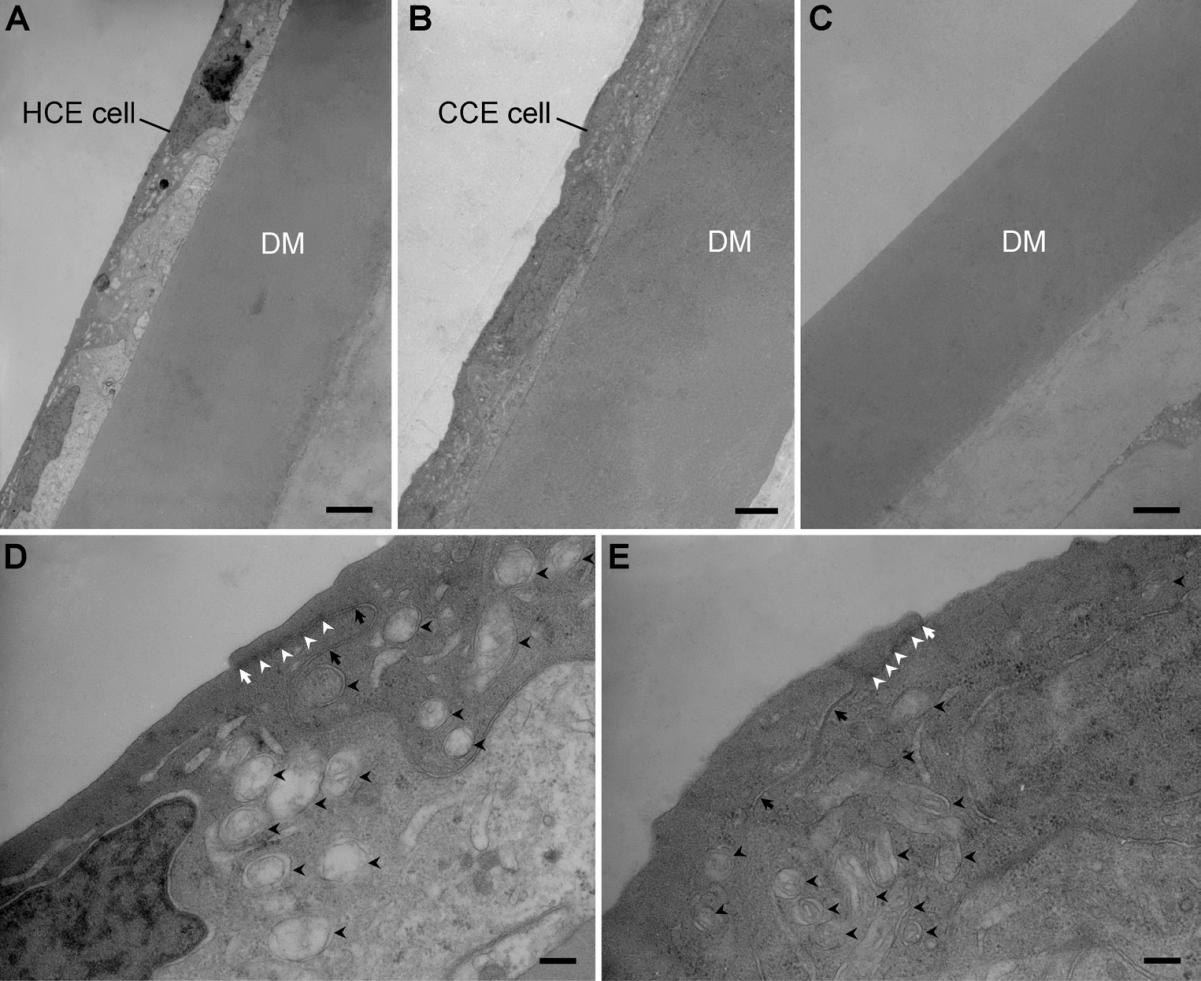
Representative transmission electron microscopy (TEM) photographs of corneas in tissue-engineered human corneal endothelium (TE-HCE) eye (**A**, **D**) normal control eye **B**, **E** and denuded amniotic membrane (dAM) eye **C** from transplanted cats. DM represents Descemet’s membrane. White arrow pointed out the intercellular tight junctions, white arrow heads pointed out the anchoring junctions such as adherens junctions and desmosomes, black arrow pointed out the gap junctions, while black arrow heads pointed out the mitochondria. Scale bars **A**, **B**, **C** 2 μm; **D**, **E** 200 nm.

Under TEM, the corneal endothelial cells of the TE-HCE eyes were observed to form a continuous monolayer endothelium with abundant intercellular junctions, including tight junctions (white arrows), anchoring junctions such as adherens junctions and desmosomes (white arrow heads), and gap junctions (black arrows) and plenty of cytoplasmic mitochondria (black arrow heads) at 104 days after transplantation (Figure 6A,D). These cells attached tightly to the remnant DM on the posterior surface of cat cornea (Figure 6A), while no corneal endothelial cells were found attached to the remnant DM in dAM eyes (Figure 6C). The monolayer status of corneal endothelium, the ultrastructure and established intercellular junctions of the corneal endothelial cells of the TE-HCE eyes were comparable to that of the normal control eyes (Figure 6B, E).

## Discussion

TE-HCEs were successfully reconstructed by using C3B strain of mcHCE cells as seeder cells and dAMs as scaffold carriers in this study. There have been reports of several methods to produce TE-HCEs, such as the use of oncogene-transfected HCE cells, primary cultured HCE cells, and limited subcultured HCE cells [8,9,14-16]. However, we employed a monoclonal strain of HCE cells, screened out from the previously established untransfected HCE cell line [17,18], as seeder cells. Moreover, the untransfected HCE cells are certainly favorable for TE-HCE reconstruction and clinical use in future; they are not tumorigenic [17]. The reconstructed TE-HCE has been only transplanted into rabbit models by now [21,22]. Since rabbits are the only animals in mammals whose corneal endothelial cells maintain proliferation [19,20], it is difficult to determine whether the transplanted TE-HCE functions by itself or cooperation with the marginal inherent rabbit corneal endothelial cells. Therefore, it is of great importance to reconstruct a high cell density TE-HCE and examine its functions after transplantation into cat models, because corneal endothelial cells from adult cats lose their proliferative ability in vivo just like the other mammals except rabbits [1,19].

Although we cultured HCE cells on dAM for only 4 days, their morphologic and structural properties were similar to HCE cells in vivo. The TE-HCE consists of a monolayer of polygonal mcHCE cells, which attached tightly to dAM and developed numerous intercellular junctions. The average cell density 4 days after in vitro reconstruction was 3,413.33±111.23 cells/mm^2^, equal to that of normal HCEs in vivo of a 10-year-old child. Therefore, it is “young” enough to be used for clinical transplantation.

Corneal transparency was recovered gradually and the average corneal thickness was 751.33±11.37 μm on day 104 after all the endothelium and part of the DM stripped and endothelial keratoplasty surgery in the TE-HCE group, whereas the corneal transplant was edematous and the average corneal thickness was 1,015.67±30.92 μm on day 104 in the dAM group. The corneal thickness of TE-HCE group was over 1,000 μm during early postoperative period, which could be explained by incomplete integration of the transplanted TE-HCE with cat corneal stroma and little or weak barrier and pump functions were performed by the cells in the transplanted TE-HCE. During late postoperative period, the transplanted TE-HCE integrated completely with cat corneal stroma and performed normal barrier and pump functions. These observations may explain why the cornea thickness decreased over time. However, there was no obvious relationship between the eye pressure of TE-HCE, dAM, and normal control eyes. All of these findings indicate that transplanted TE-HCE functions well in vivo and contributes to the maintenance of corneal clarity.

The morphology and structure of the transplanted TE-HCE were also examined in vitro. On day 104 after transplantation, a monolayer of continuous corneal endothelial cells from the transplanted TE-HCE was reconstructed. Abundant intercellular junctions, including tight junctions, adherens junctions, desmosomes, and gap junctions, were established in the TE-HCE group. The mcHCE cells were either hexagonal or polygonal in shape, and were similar to those of the normal control eyes. The cell density of corneal endothelia of the TE-HCE eyes was 2,573.33±0.59 cells/mm^2^, which was slightly lower than that of the normal control eyes (2,693.33±0.70 cells/mm^2^); however, such a density was enough to maintain corneal clarity in clinical situations after transplantation. All these findings indicate that the corneal endothelium reconstructed from the transplanted TE-HCE was almost the same in cell morphology and density as those of the normal control endothelium.

The ultrastructure of mcHCE cells in transplanted TE-HCE was similar to that of normal HCE cells in vivo at 104 days after TE-HCE transplantation. The remnant DM was covered with a monolayer of corneal endothelial cells, which attached tightly to DM. The monolayer status of the reconstructed corneal endothelium and ultrastructure of mcHCE cells in the TE-HCE eyes were almost identical to those of the normal control eyes, indicating that the corneal endothelium reconstructed from the transplanted TE-HCE was almost in the same histological structure and cellular ultra-structure as those in the normal control eye.

In conclusion, a TE-HCE in normal morphology and structure was reconstructed by in vitro culturing of mcHCE cells on dAM. After transplantation, this developed in vivo into a monolayer of continuous mcHCE cells with almost the same morphology, density, and ultrastructure as those of normal corneal endothelium. The TE-HCE functioned well and contributed to the maintenance of corneal clarity. Therefore, the reconstructed TE-HCE could function as an equivalent of corneal endothelium and restore corneal function in cats. It is useful in regenerative medicine and provides a promising method for the treatment of diseases caused by corneal endothelial disorders.
